# Postoperative intercostal lung hernia

**DOI:** 10.1002/rcr2.323

**Published:** 2018-04-27

**Authors:** Akane Ishida, Masahide Oki, Hideo Saka, Yukio Seki

**Affiliations:** ^1^ Department of Respiratory Medicine Nagoya Medical Center Nagoya Japan; ^2^ Department of Thoracic Surgery Nagoya Medical Center Nagoya Japan

**Keywords:** Complications, lung hernia, thoracotomy, Valsalva manoeuvre

## Abstract

Lung hernia is a rare condition defined as the external protrusion of lung tissue from the thorax, which usually occurs after trauma or thoracic surgery. A chest computed tomography while performing the Valsalva manoeuvre is useful for a definitive diagnosis.

## Clinical Image

**Figure 1:**
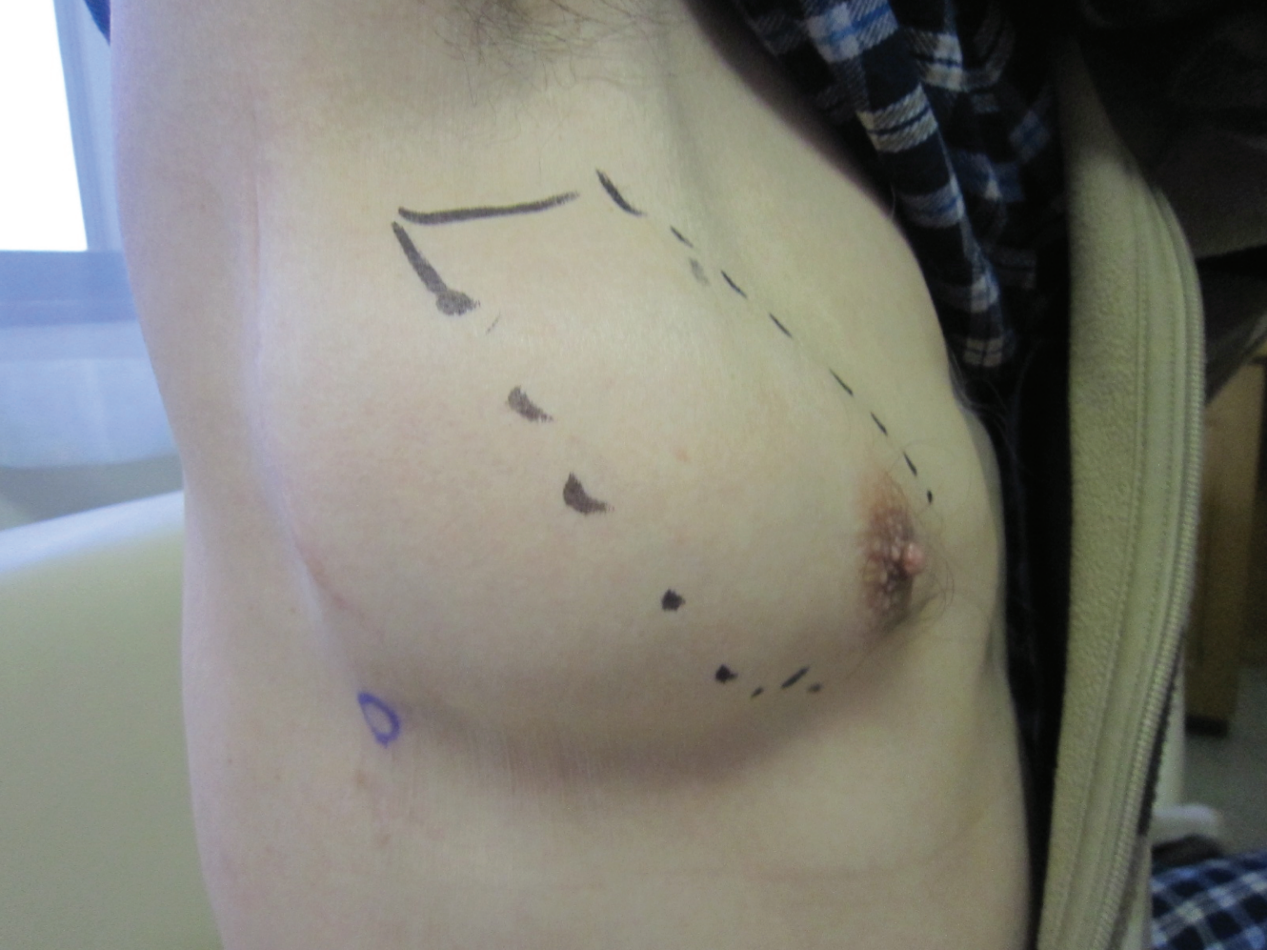
A bulge on the anterolateral chest wall on coughing.

**Figure 2:**
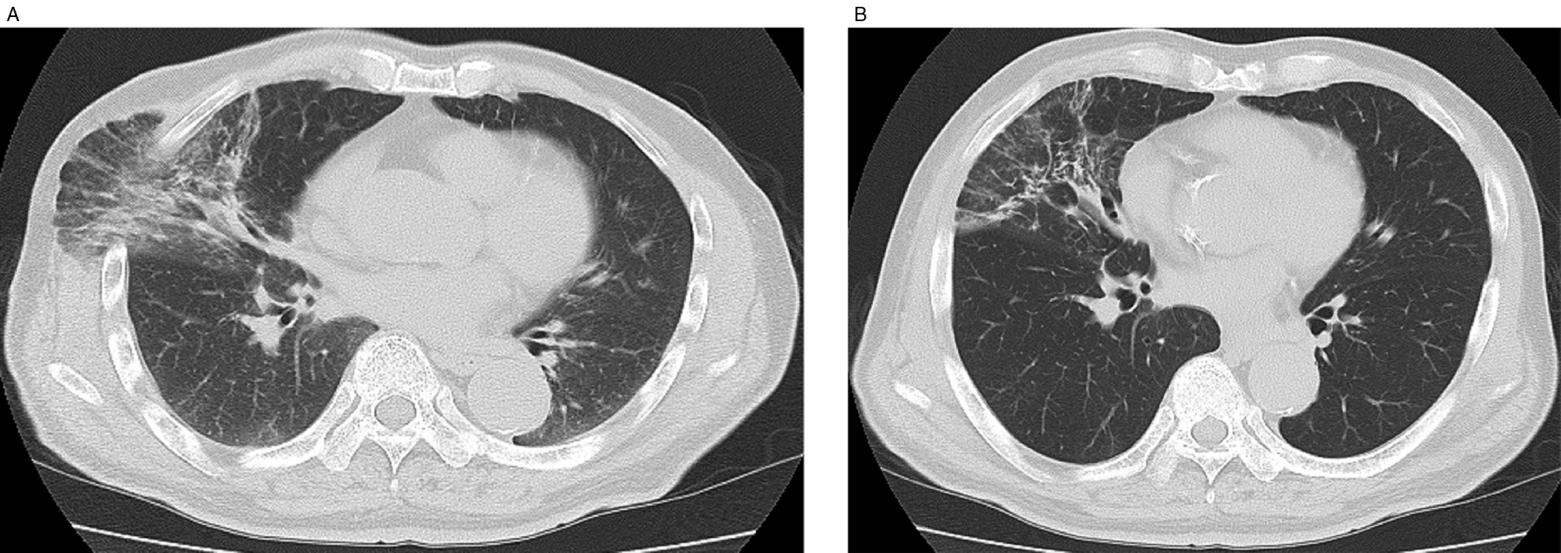
(A) Chest computed tomography (CT) during Valsalva manoeuvre. Lung tissue with inflammation is protruding beyond the rib cage. (B) Chest CT at mid‐inspiration.

**Figure 3:**
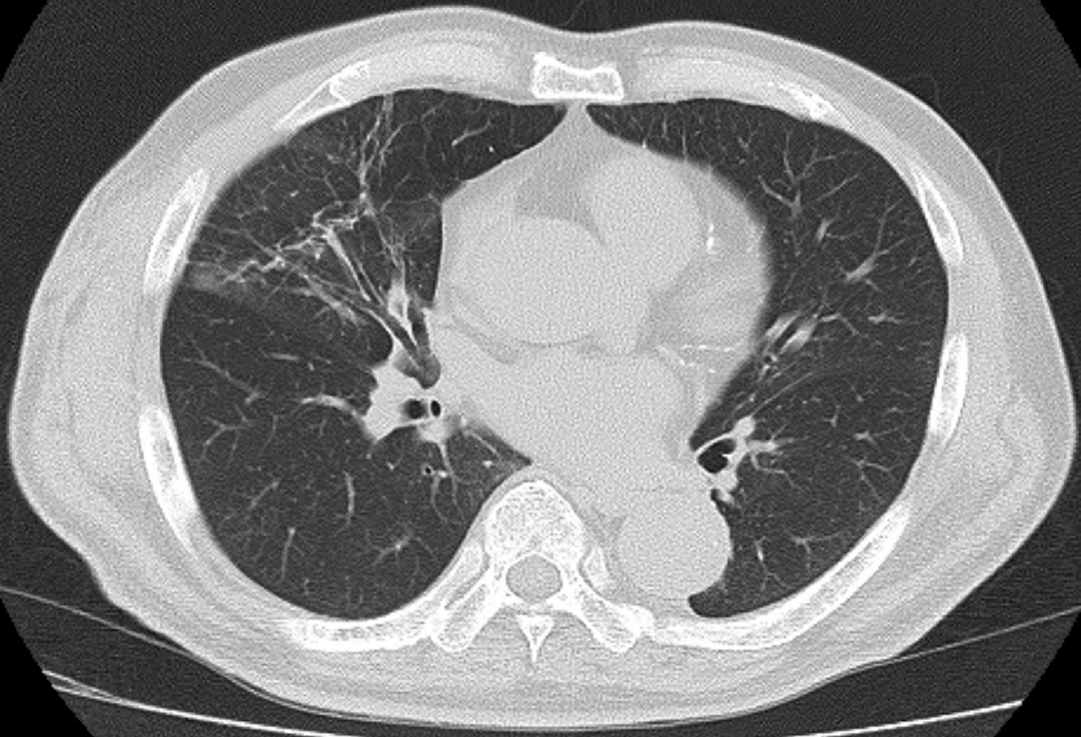
Chest computed tomography (CT) 1 year after surgical repair.

A 73‐year‐old man received positron emission tomography‐computed tomography (CT) screening to detect malignant disease, which revealed a hyper‐metabolic lesion in the right hilar lymph node. He underwent open thoracotomy for lymph node sampling, and the result was non‐specific lymphadenopathy. Twenty months after the thoracotomy, he had a cough and became aware of a protrusion from the intercostal space during coughing. Two years after the operation, the size of the protrusion increased, and a chest X‐ray revealed abnormalities, so he was referred to our hospital for examination and treatment. Physical examination showed skin protruding anterior laterally from the fourth right intercostal space during the Valsalva manoeuvre (Fig. 1). Chest CT revealed protruding lung tissue with inflammation from the intercostal space (Fig. 2A, B). Due to the increasing size of the bulge, the persistent cough, and inflammatory changes in the protruding lung, surgical repair was performed. A polypropylene surgical mesh was implanted into the sub‐pleural space after plication of the hernia sac, and the musculature of the thoracic wall was performed. No recurrence was detected one year later, and chest CT showed amelioration of inflammation in the protruded lung (Fig. 3).

Lung hernia is a rare condition defined as external protrusion of lung tissue from the thorax and is caused by elevated intra‐thoracic pressure and defects or weakness in the thoracic wall. Most cases are acquired, usually from trauma or thoracic operation 1. Postoperative lung herniation has been reported to occur more commonly after less extensive thoracic surgeries, such as mini‐thoracotomy and video‐associated thoracoscopy, compared to major thoracic surgeries 1. The reason for this is that the pericostal closure through the smaller skin incision in minor thoracic surgeries may be less meticulous. A meticulous pericostal closure likely prevents the occurrence of lung hernia after major thoracic surgeries. In anatomical classification, cervical or diaphragmatic herniation is less common than intercostal herniation. For diagnosis, a chest CT while performing the Valsalva manoeuvre is recommended 1, 2. Increasing size, the presence of symptoms, and the presence of incarceration are indications for surgical repair 1.

## Disclosure Statement

Appropriate written informed consent was obtained for publication of this case report and accompanying images.
